# Helminth-derived molecules: Pathogenic and pharmacopeial roles

**DOI:** 10.7555/JBR.38.20240177

**Published:** 2024-09-24

**Authors:** Yu Zhang, Chunxiang Shen, Xinyi Zhu, Chiuan Yee Leow, Minjun Ji, Zhipeng Xu

**Affiliations:** 1 State Key Laboratory of Reproductive Medicine and Offspring Health, Department of Pathogen Biology, National Vaccine Innovation Platform, Nanjing Medical University, Nanjing, Jiangsu 211166, China; 2 School of Pharmaceutical Sciences, Universiti Sains Malaysia, Minden, Penang 11800, Malaysia; 3 NHC Key Laboratory of Antibody Technique, Nanjing Medical University, Nanjing, Jiangsu 211166, China

**Keywords:** helminth, helminth-derived molecules, immunity, metabolism, excretory/secretory products, extracellular vesicles

## Abstract

Parasitic helminths, taxonomically comprising trematodes, cestodes, and nematodes, are multicellular invertebrates widely disseminated in nature and have afflicted humans continuously for a long time. Helminths play potent roles in the host by generating a variety of novel molecules, including some excretory/secretory products and others that are involved in intracellular material exchange and information transfer as well as the initiation or stimulation of immune and metabolic activation. The helminth-derived molecules have developed powerful and diverse immunosuppressive effects to achieve immune evasion for parasite survival and establish chronic infections. However, they also improve autoimmune and allergic inflammatory responses and promote metabolic homeostasis by promoting metabolic reprogramming of various immune functions, and then inducing alternatively activated macrophages, T helper 2 cells, and regulatory T cell-mediated immune responses. Therefore, a deeper exploration of the immunopathogenic mechanism and immune regulatory mechanisms of helminth-derived molecules exerted in the host is crucial for understanding host-helminth interactions, as well as the development of therapeutic drugs for infectious or non-infectious diseases. In this review, we focus on the properties of helminth-derived molecules to give an overview of the most recent scientific knowledge about their pathogenic and pharmacopeial roles in immune-metabolic homeostasis.

## Introduction

Throughout history, humans have always struggled to combat infectious diseases that are also commonly referred to as communicable diseases. It is worth mentioning that parasitic diseases remain one of the most important infectious diseases around the world. Globally, there are more than two billion individuals infected with helminths, which is a significant public health problem ^[ 1] ^. Helminth infections lead to substantial tissue damage and then result in chronic malnutrition, impaired growth, and increased vulnerability to deadly diseases as they migrate through different organs to complete their life cycles ^[ 2] ^.

A co-evolutionary adaptation between helminths and the host often occurs through immune interactions. During helminth invasion, the host may injure, contain, and/or expel helminths through a constantly adaptive immune response. Meanwhile, as a result of co-adapting with their hosts and coping with the host-induced immune responses during infection, helminths have developed subtle immune regulatory mechanisms (mainly the type 2 immune response), including the differentiation of a cluster of differentiation 4 (CD4) ^+^ T cells towards a T helper 2 (Th2)/regulatory T (Treg) phenotype and the polarization of macrophages towards an alternatively activated macrophage (M2) phenotype, to evade the host, protect themselves, and establish a chronic infection that is beneficial for reducing inflammation and repairing damaged tissues ^[ 3] ^. Additionally, helminths also rely on a wide variety of metabolic enzymes to decompose host metabolites or nutrients to maintain their growth, while minimizing host tissue immunopathology simultaneously ^[ 4] ^. Therefore, this state of co-evolutionary cross-talk between helminths and their hosts primarily depends on the precise regulation of the host immunity and metabolism.

Inverse associations between helminth infection rates and the prevalence of immune-related diseases have been found by epidemiological investigations, leading to the emergence of a novel therapeutic strategy called helminth therapy that implies a therapeutic potential of worms in a variety of autoimmune and inflammatory diseases, such as asthma, inflammatory bowel disease, and rheumatoid arthritis ^[ 5] ^. Investigators have hypothesized that helminth infections restrict the inflammation correlated with non-helminth infections, such as the severe acute respiratory syndrome coronavirus 2 (SARS-CoV-2) and influenza A virus, leading to a remission in clinical outcomes ^[ 6– 7] ^. However, to support this claim, more epidemiological studies on the alleviation of helminth-mediated virus infection are required ^[ 8] ^. Based on this natural co-evolutionary feature, helminth therapy possibly has a lower side effect risk, compared with conventional medications, and provides new options for people who cannot take current medications for their special conditions ^[ 9] ^. Every coin has two sides, and there is no exception that the natural treatment of helminth therapy also has risks and side effects, because some kinds of helminths may worsen diseases like asthma; hence, it is imperative to figure out which helminths are beneficial or harmful ^[ 10– 11] ^.

Because of the complex immunoregulation and safety issues, helminth infection is not considered the best treatment option for inflammation and metabolism-related diseases. The rapid and in-depth development of multi-omics techniques has inspired researchers to identify helminth-derived molecules and investigate their biological functions and mechanisms, making the regulation of host immunity and metabolism by helminth-derived molecules an important research area in recent years ^[ 12] ^. Helminth-derived molecules, including excretory/secretory proteases, proteins, miRNAs, lipids, glycans, extracellular vesicles (EVs), and other small molecules, such as short-chain fatty acids and amino acids, have been isolated and identified gradually ^[ 13– 14] ^. On the one hand, these molecules enable helminths to successfully migrate and develop strategies to complete their life cycles ^[ 15] ^. On the other hand, the interaction of helminth-derived molecules with the host induces stable anti-inflammatory immune responses, which is beneficial for improving the immunopathology of the parasitic site in the host, and in the meantime, these molecules also show various biological and pharmacological activities in other diseases ^[ 16] ^.

As a subcategory of excretory/secretory products (ESPs), the helminth-derived EVs carry a variety of functional proteins, lipids, RNA, glycans, and other molecules, and may transfer these bioactive molecules from parasites to target cells of the host to mediate the immunity and metabolism communication between the invader and innate immune cells ^[ 17] ^. The EV-loaded components, even at different stages of the development, have been gradually identified and investigated with the help of multi-approach ^[ 18– 19] ^. For example, the EV-loaded and transported miRNAs show commonalities and characteristics in the *Schistosoma mansoni* ( *S. mansoni*, Sambon, 1907) and *Fasciola hepatica* ( *F. hepatica*, Linn, 1758) infections, which may act as potential biomarkers for the diagnosis of trematode infections and also hold a great promise for the distinction of different trematode infections ^[ 20] ^. According to the accumulating evidence, the helminth-derived EVs also display a robust potential to act as new targets for vaccines and other novel therapies, which hold a promise in the prevention and control of parasitic diseases, such as how best to present EV antigens and how to design vaccination strategies ^[ 21] ^.

In this review, the deeper mining of the composition, characteristics, and functions of these helminth-derived molecules will not only enable us to understand the pathogenic effect on the host, but also from a therapeutic view to lay a foundation for relevant vaccines and immunotherapy for infectious or non-infectious diseases based on their regulatory effects. Finally, despite the quick and thorough development of multi-omics tools to find exceptional compounds, there are still many unknown helminth-derived molecules awaiting identification, most of which are currently in a mixed form, for which we have gathered these resources as antigens in this review.

## Schistosome-derived molecules

### Proteases/protease inhibitors/redox enzymes

Parasite proteases are important virulence factors in many parasite infections and may be key molecules in host-parasite interactions. To protect themselves from host enzyme degradation, parasites also release proteinase inhibitors that help regulate host tissue damage and exhibit potential immunomodulatory effects, playing a key role in maintaining homeostasis ^[ 22] ^. Considering that cercariae invasion of the human skin is the first step in schistosome infection, Zhu *et al* ^[ 23]^ demonstrated the facilitative role of SjCB2 (a cathepsin B cysteine protease derived from cercariae) in the skin invasion process of *Schistosoma japonicum* ( *S. japonicum*, Katsurada, 1904) cercariae. In addition, cystatin (cysteine protease inhibitor) secreted by *S. japonicum* (Sj-Cys), which was expressed in the gut and tegument of adult worms (as well as in eggs), might be applied as a therapeutic agent to treat inflammatory diseases such as sepsis by activating M2 macrophage polarization ^[ 24] ^. Furthermore, Sj-Cys also mitigated sepsis-induced cardiomyopathy by inhibiting the lipopolysaccharides (LPS)-myeloid differentiation primary response 88 (MYD88) inflammatory signaling pathway and might have some therapeutic efficacy in the sepsis-associated cardiac dysfunction ^[ 25] ^. In the acute kidney injury induced by the acute liver failure model, Sj-Cys presented a protective effect against it by inhibiting pyroptosis and the nuclear factor kappa-B (NF-κB) signaling pathway ^[ 26] ^. In addition to attenuating these inflammatory diseases, Sj-Cys attenuated metabolic diseases and atherosclerosis like atherosclerotic renal damage through promoting Treg and M2 macrophage polarization, mediated by inhibiting the Toll-like receptor 2 (TLR2)/MYD88 signaling pathway ^[ 27] ^. Interestingly, in the mouse model of bone metabolic disorders, Sj-Cys also showed a protective role by inhibiting the NF-κB signaling pathway that was crucial for the early stages of osteoclastogenesis ^[ 28] ^. Overall, the immunomodulatory function of Sj-Cys mostly involves promoting M2 and Treg cells, which is dependent on the suppression of the NF-κB signaling pathway.

Neutrophils play significant roles in the inflammatory response by releasing neutrophil elastase, a kind of serine protease enzyme involved in microbicidal activity ^[ 29] ^. The multigene family of Kunitz-type inhibitors is a class of serine protease inhibitors, which has been proven to be potent neutrophil elastase inhibitors ^[ 30] ^. The *S. mansoni* Kunitz-type serine protease inhibitor (SmKI-1) detected in larval and adult phases conferred an anti-inflammatory effect in acetaminophen-mediated liver damage, gout arthritis, and the carrageenan-induced pleurisy model by inhibiting the activity of neutrophil elastase and neutrophil inflammatory infiltration ^[ 30] ^. Furthermore, SmKI-1, in a recombinant form, maintained a Th1/Th2-type balanced protective response and produced a solid IgG response, demonstrating a vaccine potential of SmKI-1 against *S. mansoni* ^[ 31] ^.

To survive within their hosts, parasites also secrete a variety of redox enzymes that are proposed to largely play roles in detoxifying oxygen radicals produced by infection-stimulated host phagocytes and controlling oxidative stress around the worms ^[ 32] ^. These redox enzymes probably include glutathione S transferases, thioredoxins, thioredoxin peroxidases (TPxs), *etc*. For instance, recombinant *S. japonicum* thioredoxin peroxidases (rTPxs) exerted anti-inflammatory effects by triggering the production of M2 macrophages in response to LPS stimulation, which may represent a potential vaccine candidate and/or new drug target against *S. japonicum* infection ^[ 33– 34] ^.

### Proteins/peptides

Heat shock proteins (HSPs) are stress proteins widely distributed in parasites. Studies have revealed the significance of several schistosome-derived HSPs in the diagnosis and regulation of hepatic immunopathology. For instance, both *S. japonicum* HSP40 (Sjp40) and Sjp90α secreted from eggs into host tissues significantly induced host innate and adaptive immune responses ^[ 35] ^. The recombinant Sjp40 (rSjp40) stimulated the production of macrophages (MΦ), dendritic cells (DCs), and eosinophilic granulocytes (ECs) in non-parenchymal mouse liver cells, and the rSjp40 stimulation *in vitro* promoted the Th1, Th2, and Th17 immune responses, but rSjp90α stimulated only the production of DCs in non-parenchymal mouse liver cells and the rSjp90α stimulation *in vitro* induced only the Th17 immune response ^[ 35] ^. Of note, our laboratory demonstrated the metabolic regulatory potential of Sjp40 in obesity-related fatty liver, mediated by activating the AMP-activated protein kinase (AMPK) pathway, and subsequently inhibiting lipogenesis by interacting with CD36 on hepatocytes to inhibit miR-802 expression ^[ 36] ^. The rSjp60 induced Tregs through the TLR4-MYD88 adaptor-like (Mal)-derived production of transforming growth factor-β (TGF-β) in macrophages but reduced liver immunopathology in mice with *S. japonicum* infection ^[ 37] ^. In addition, the Sjp60-derived peptide SJMHE1 exerted a protective effect on autoimmune diseases, such as colitis, collagen-induced arthritis, and ovalbumin-induced delayed-type hypersensitivity, by re-establishing immunological homeostasis, including the decrease of Th1/Th17 cells and the increase of Th2/Treg cells ^[ 38– 40] ^. Similar anti-inflammatory and anti-fibrotic effects in schistosomiasis were also observed from recombinant *S. japonicum* secreted-protein 16 (rSj16), which may also be anticipated to serve as a potential source for the medication of other inflammatory diseases like dextran sodium sulfate (DSS)-induced colitis in the mouse model ^[ 41] ^.

Beyond direct regulation of the immune-metabolic homeostasis, the helminth-derived molecules achieve indirect regulation of the host immune-metabolic homeostasis through the hepatic stellate cells (HSCs)-immune interactions. HSCs become dysfunctional because of inflammation, and their activation, characterized by the increased expression levels of α-smooth muscle actin (α-SMA) and collagen 1α1 (COL1α1), contributes to the granulomatous and hepatic fibrosis induced by schistosomiasis eggs ^[ 42] ^. TGF-β1 is the most potent fibrogenic cytokine for HSC activation, and the regulatory roles of *S. japonicum*-derived molecules on hepatic fibrosis mainly target the TGF-β1, interleukin-34 (IL-34), and HSCs ^[ 43] ^. It has been reported that Sjp40 was therapeutically effective in the hepatic fibrosis model by preventing the activation of HSCs *in vitro* and suppressing the TGF-β and extracellular signal-regulated kinase signaling pathways ^[ 44] ^. Further investigation demonstrated that SjP40 inhibited the expression of COL1α1 in the activated HSCs to prevent the excessive deposition of extracellular matrix molecules in the liver by promoting the expression of transcription factor ETS proto-oncogene 1 ^[ 45] ^. To investigate the effect of *S. mansoni* Sm16 on hepatic fibrosis, researchers compared two synthetic peptides of Sm16 (KS-84 and KS-66) and discovered that KS-84 inhibited HSC activation by reducing the TGF-β1 signaling, while KS-66 enhanced the activation of HSCs; the cause of this disparity remained to be further investigated ^[ 46] ^.

### miRNAs

Based on the above description, targeting the molecules derived from *S. japonicum* may be an important therapeutic strategy for treating liver diseases caused by schistosomiasis infection. Among these molecules, miRNAs play important roles in regulating host gene expression and cell phenotype, promoting helminth development and immune regulation/evasion as well as mediating various pathological processes associated with helminth infection, which may be used as stimulators for the host-parasite interaction and diagnostic biomarkers for helminth infection ^[ 47] ^. An miRNA derived from *S. japonicum* was identified and termed miR-2162-3p that was consistently present in HSCs from the *S. japonicum*-infected mice, and directly targeted and downregulated the expression level of TGF-β receptor Ⅲ, a known TGF-β signaling negative regulator, to promote the activation of HSCs and hepatic fibrosis in the host ^[ 48] ^. The WNT/β-catenin pathway plays a pivotal role in activating HSCs and promoting the expression of TGF-β ^[ 49] ^. The *S. japonicum*-derived miRNA Sja-miR-1 was reported to upregulate the expression levels of collagens and α-SMA and promote hepatic fibrosis by inhibiting the expression of secreted frizzled-related protein 1, a negative regulator of the WNT/β-catenin pathway ^[ 50] ^. These studies reveal that the inhibition of *S. japonicum*-derived miRNAs may alleviate the hepatic pathological process. Additionally, Sja-miR-7-5p, an miRNA derived from *S. japonicum*, has been shown to exhibit antitumor activity against hepatocellular carcinoma during schistosome infection by inhibiting the expression of S-phase kinase-associated protein 2 that promotes the viability and migration of tumor cells ^[ 51] ^.

### EVs/EV-loaded molecules

The EVs derived from adult *S. japonicum* were mainly taken up by the host peripheral cells and then transferred their cargo miRNAs into the recipient cells ^[ 52] ^. It was reported that adult *S. japonicum* EVs-derived miR-125b and bantam miRNA regulated the corresponding targets (including prostate-specific antigen 1, family with sequence similarity 212 member B, and CXADR-like membrane protein) to promote the proliferation of macrophages and the production of tumor necrosis factor-alpha (TNF-α) ^[ 52] ^. Investigators also identified a novel miRNA (miRNA-33) derived from the *S. japonicum* egg-released EVs that upregulated the expression levels of α-SMA and COL1α1 in liver tissues and caused hepatic fibrosis in the host through the TGF-β/small mother against decapentaplegic family member 3 (SMAD3) signaling pathway, suggesting that inhibiting the expression of this miRNA may alleviate the degree of fibrosis ^[ 53] ^. In contrast, Sja-miR-71a derived from the *S. japonicum* egg-released EVs was reported to suppress the pathological progression of hepatic fibrosis by inhibiting the TGF-β1/SMAD and IL-13/signal transducer and activator of transcription 6 (STAT6) pathways *via* directly targeting semaphorin 4D ^[ 54] ^. During *S. japonicum* infection, Sja-miR-3096 derived from the *S. japonicum* egg-released EVs was present in the hepatocytes of mice and mediated antitumor activity in liver cancer through targeting phosphoinositide 3-kinase class Ⅱ alpha, which may offer potential cancer therapeutic strategies based on the capacity of such helminth derived-miRNAs ^[ 55] ^.

Meningher *et al* ^[ 56]^ discovered that adult *S. mansoni* EVs were internalized by Th cells, and that the miR-10 contained in these EVs might downregulate the NF-κB activity by targeting mitogen-activated protein kinase 7 (MAPK7) in Th cells. Despite the identification of many helminth EV-loaded proteins and miRNA, little is known about the surface glycans of EVs in their interaction with host cells. The DC-specific C-type lectin (DC-SIGN) that acts as a pathogen receptor for *S. mansoni* egg antigens recognizes and binds to the glycan antigen Lewis x ^[ 57] ^. Investigators discovered that glycosylated *S. mansoni* schistosomula EVs might be internalized by DCs *via* glycan-mediated binding to DC-SIGN, while enhancing the expression of IL-12 and IL-10 in DCs ^[ 58] ^. Further glycomics studies on adult *S. mansoni* EVs demonstrated that the glycan structures with sialic acid residues on the surface of adult *S. mansoni* EVs adhered to *Sambucus nigra* lectin-Ⅰ, a lectin-recognizing terminal sialic acid structure ^[ 59] ^. Therefore, adult *S. mansoni* EVs may participate in immune evasion by being coated with sialoglycans *in vivo* or being encapsulated by exogenous host sialylated molecules, preventing their clearance from the serum and contributing to cell adhesion as well as entering the host to exert the function ^[ 60] ^. In addition, one study found that the protein and lipid composition of plasma circulating EVs in hamsters infected with *S. mansoni* was different from that of uninfected hamsters, suggesting that *S. mansoni* infection also had a significant effect on the composition of circulating EVs in the host plasma ^[ 61] ^.

### Antigens

**Figure 1:**
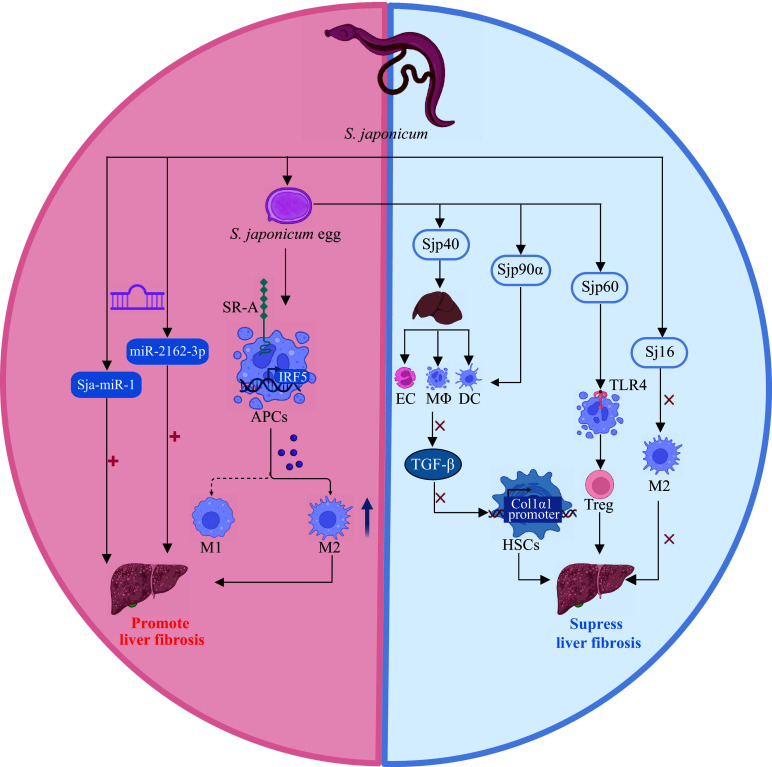
Immune and metabolic regulation by the *S. japonicum*-derived molecules. Abbreviations: Sj16, *S. japonicum* secreted-protein 16; miR-2162-3p/Sja-miR-1, *S. japonicum* derived-miRNA; DC, dendritic cells; HSCs, hepatic stellate cells; SR-A, scavenger receptor A; M1, classically activated macrophages; M2, alternatively activated macrophages; APCs, antigen-presenting cells; COL1α1, collagen type 1 alpha 1; Sjp40/90α/60, *S. japonicum* heat shock proteins 40/90α/60; TGF-β, transforming growth factor-beta; Treg, regulatory T cell; TLR4, Toll-like receptor 4; EC, eosinophilic granulocyte; MΦ, macrophage.

During schistosome infection, female helminth-released soluble egg antigen (SEA) favorably stimulated the M2 phenotype. This stimulation was mediated by promoting the endocytosis of the pattern recognition receptor scavenger receptor A on the macrophage cell membrane into the cytoplasm and by suppressing the nuclear transcription of interferon regulatory factor 5 (IRF5) through binding with IRF5 in the cytoplasm, which inhibited M1 (classically activated macrophages) polarization and promoted M2 and Th2 polarization, and finally participated in the formation of hepatic immunopathology ^[ 62] ^. In addition to mediating a series of inflammatory responses to facilitate the formation of egg granulomas and hepatic fibrosis, both *S. japonicum* and *S. mansoni*-derived SEA may exert therapeutic effects on hepatic fibrosis and various pro-inflammatory models (such as type 2 diabetes, skin transplantation rejection models, and wound models) by inhibiting Th1/Th17 immune responses and enhancing Th2/Treg immune responses ^[ 63– 66] ^. The restoration of Treg/Th17 balance by *S. japonicum* SEA was associated with the inhibition of glycolysis pathway and lipogenesis, which played a protective role against trinitrobenzene sulfonic acid (TNBS)-induced colitis ^[ 67] ^. Additionally, the ESPs from *S. japonicum* eggs were also found to alleviate ovalbumin-induced allergic airway inflammation ^[ 68] ^. Finally, we briefly summarize the pathogenic and pharmacopeial roles of *S. japonicum*-derived molecules in liver fibrosis ( ***Fig. 1***).

## Other trematode-derived molecules

### Proteases/protease inhibitors/redox enzymes

*Clonorchis sinensis* ( *C. sinensis*, Cobbold, 1875) is a carcinogenic human liver fluke endemic in South China, Japan, Korea, *etc.*, which induces cholangitis, epithelial hyperplasia, periductal fibrosis, and even cholangiocarcinoma (CCA) in the host. Following the extensive knowledge of the immunological mechanisms involved in the pathogenicity of *C. sinensis* ESPs, vaccination, the best strategy to prevent and control clonorchiasis, has been investigated from the *C. sinensis*-derived molecules. For example, *C. sinensis*-derived cysteine proteases were demonstrated to facilitate Th2/Treg immune responses, which promoted the development of vaccination and potential therapeutic targets against some autoimmune diseases ^[ 69] ^.

*F. hepatica* is a zoonotic parasitic helminth common in temperate and tropical regions globally and is a considerable economic and public health problem worldwide. Cathepsin L-like cysteine peptidases derived from *F. hepatica* were demonstrated to induce the alternative activation of NLR family pyrin domain containing 3 inflammasome in murine DCs and the production of pro-inflammatory cytokines (IL-1β and IL-18) ^[ 70] ^. In addition to secreting an abundance of cathepsin peptidases to facilitate invasion and migration, *F. hepatica* also secreted various regulatory protease inhibitors. For example, De Marco Verissimo *et al* ^[ 71]^ demonstrated that the existence of serine protease inhibitors in *F. hepatica* ESPs protected the invading parasites against the harmful proteolytic effects of host proteases to promote the survival of parasites. The *F. hepatica* Kunitz-type serine protease inhibitor (FhKTM) presented in both the gut and tegument of the adult worm regulated the host inflammatory responses by promoting Th2 immune responses and impairing Th1/Th17-related inflammation responses ^[ 72] ^. Additionally, cystatin secreted by *F. hepatica* (FhCystatin) regulated the activity of macrophages, participated in immune evasion, and prevented excessive damage to both parasite and host tissues ^[ 73] ^. Cwiklinski *et al* ^[ 74]^ presented a vaccine strategy to disrupt the protease/anti-protease balance of parasites by targeting key peptidase inhibitors to prevent and control *F. hepatica* infection.

In a previous study, the *F. hepatica*-derived molecules including some redox enzymes involved in parasite defense against the host immune system, such as superoxide dismutase and glutathione S-transferases, were identified through a proteomic approach ^[ 75] ^. The native glutathione S-transferase secreted by *F. hepatica* (nFhGST) had anti-inflammatory effects on the host, which might be mediated by targeting multiple effectors of NF-κB, the Janus kinase (JAK)/STAT signaling, and other pathways as well as inhibiting the production of pro-inflammatory cytokines/chemokines ^[ 76– 77] ^. A novel glutathione S-transferase Omega 2 secreted by *F. hepatica*, which was mainly expressed in the vitelline follicles, intestinal tract, excretory pores, and vitelline cells in *F. hepatica*, might be taken up by host macrophages and played a vital role in promoting apoptosis and inhibiting the production of pro-inflammatory cytokines, which may drive the development of anti-inflammatory agents based on its immunoregulatory activity ^[ 78] ^.

### Proteins/peptides

*C. sinensis*-derived proteins, such as HSP70/90 proteins, induced strong Th1 immune responses and promoted the secretion of pro-inflammatory cytokines in the mouse biliary epithelial cells (BECs), promoting the progression of clonorchiasis ^[ 79– 80] ^. In turn, the antigenic protein CsAg17 secreted by *C. sinensis*alleviated the helminth burden and had the potential to serve as a good vaccine candidate against *C. sinensis* infections ^[ 81] ^.

One study investigated the potential of peptide-helminth defense molecule 1 (FhHDM-1) secreted by *F. hepatica* in inflammatory diseases, and found that it prevented the development of type 1 diabetes in non-obese diabetic mice by activating the phosphatidylinositol-3-kinase/protein kinase B (PI3K/AKT) pathway and preserved β-cell mass/function ^[ 82] ^. Compared with other PI3K/AKT pathway regulators, FhHDM-1 not only enhanced the function of pancreatic β-cells and prevented apoptosis but also inhibited carcinogenesis caused by excessive proliferation of pancreatic β-cells, which had significant advantages in treating type 1 diabetes in mice ^[ 83] ^. Therefore, the development of helminth-derived molecules for the treatment of autoimmune diseases may have more potential than traditional new drug development methods based on the co-adaptive characteristics formed during the co-evolution of helminths their hosts.

### EVs/EV-loaded molecules

Liver fluke EVs play crucial roles in biliary injuries, cholangitis, biliary fibrosis, and possibly CCA induced by chronic infection with liver flukes. For instance, miRNA Csi-let-7a-5p derived from adult *C. sinensis* EVs promoted the M1 polarization and contributed to the biliary injuries by targeting the suppressor of cytokine signaling 1- and C-type lectin domain family 7 member A-mediated NF-κB signaling pathway in mice, which implied a mechanism contributing to biliary injuries induced by fluke infection from the point of helminth-derived molecules ^[ 84] ^. It has been evidenced that ferroptosis is inhibited in cancer cells, enabling them to proliferate and endure ^[ 85] ^. Furthermore, adult *C. sinensis* EVs-loaded with Csi-miR-96-5p inhibited ferroptosis by regulating the phosphatase and tensin homolog/solute carrier family 7 member 11/glutathione peroxidase 4 axis, thereby promoting CCA proliferation and migration ^[ 86] ^.

Chaiyadet *et al* ^[ 87]^ found that the adult *Opisthorchis viverrini* ( *O. viverrini*, Robert Thomson Leiper, 1915) EVs in host bile specimens during infection promoted the proliferation of bile duct cells and the secretion of IL-6 to exert a tumorigenic phenotype. Moreover, the surface of adult *O. viverrini* EVs was rich in the CD63-like tetraspanin 2 ( *O. viverrini*-tetraspanin-2, Ov-TSP-2) and Ov-TSP-3 that served as biomarkers of *O. viverrini* EVs ^[ 87] ^. It was also reported that silencing the expression of *Ov-tsp-2* and *Ov-tsp-3* genes using RNA interference technology led to a decrease in the secretion of adult *O. viverrini* EVs and a lack of their respective TSPs in the secreted part of the EVs, therefore suggesting the important roles of TSPs in maintaining the stability of adult *O. viverrini* EVs ^[ 87] ^. Additionally, adult *O. viverrini* EVs and EVs-derived recombinant tetraspanin 2 (recombinant- *O. viverrini*-tetraspanin-2, rOv-TSP-2) and rOv-TSP-3 might be used as vaccines to induce antibody responses, thus blocking the EVs from being phagocytized by bile duct cells and reducing the parasite burden ^[ 88] ^. To further understand the protective effect of anti-Ov-TSP-2 antibody against opisthorchiasis, Phumrattanaprapin *et al* ^[ 89]^ transferred two anti-Ov-TSP-2 monoclonal antibodies, 1D6 and 3F5, to hamsters at one day before parasite infection, and found that 1D6 and 3F5 might block the uptake of fluke EVs by BECs through combining with host BECs. These studies further demonstrate the efficacy of antibodies targeting adult *O. viverrini* EVs, which lay the foundation for the development of a vaccine against opisthorchiasis and fluke infections.

### Antigens

Numerous studies have shown that *C. sinensis* ESPs play pathogenic roles in the progression of hepatic fibrosis and CCA. *C. sinensis* ESPs have been reported to promote the activation of HSCs and the pathogenesis of hepatic fibrosis *via* TLR4 and the TGF-β/SMADs signaling pathway, exhibiting the crucial role of TLR4 in host defenses against *C. sinensis* and the pathogenesis of clonorchiasis ^[ 90] ^. However, a recent study revealed that *C. sinensis* ESPs promoted the production of IL-6 in mouse BECs through the TLR2-mediated AKT and p38 pathways, resulting in the aggravation of hepatic fibrosis in mice ^[ 91] ^. Considering that genetic deletion of either TLR2 or TLR4 alone significantly suppressed the *C. sinensis* ESPs-induced hepatic fibrosis according to the existing studies ^[ 91– 92] ^, we speculate that TLR2 and TLR4 appear to be synergistic promoters in this process. Additionally, it was also reported that *C. sinensis* ESPs played important roles in promoting the progression of CCA through the interaction between normal cholangiocytes and CCA cells, which might be mediated by the extracellular signal-regulated kinase 1/2/NF-κB/matrix metalloproteinase-9 and integrin β4-focal adhesion kinase/steroid receptor coactivator pathways ^[ 93– 94] ^.

**Figure 2:**
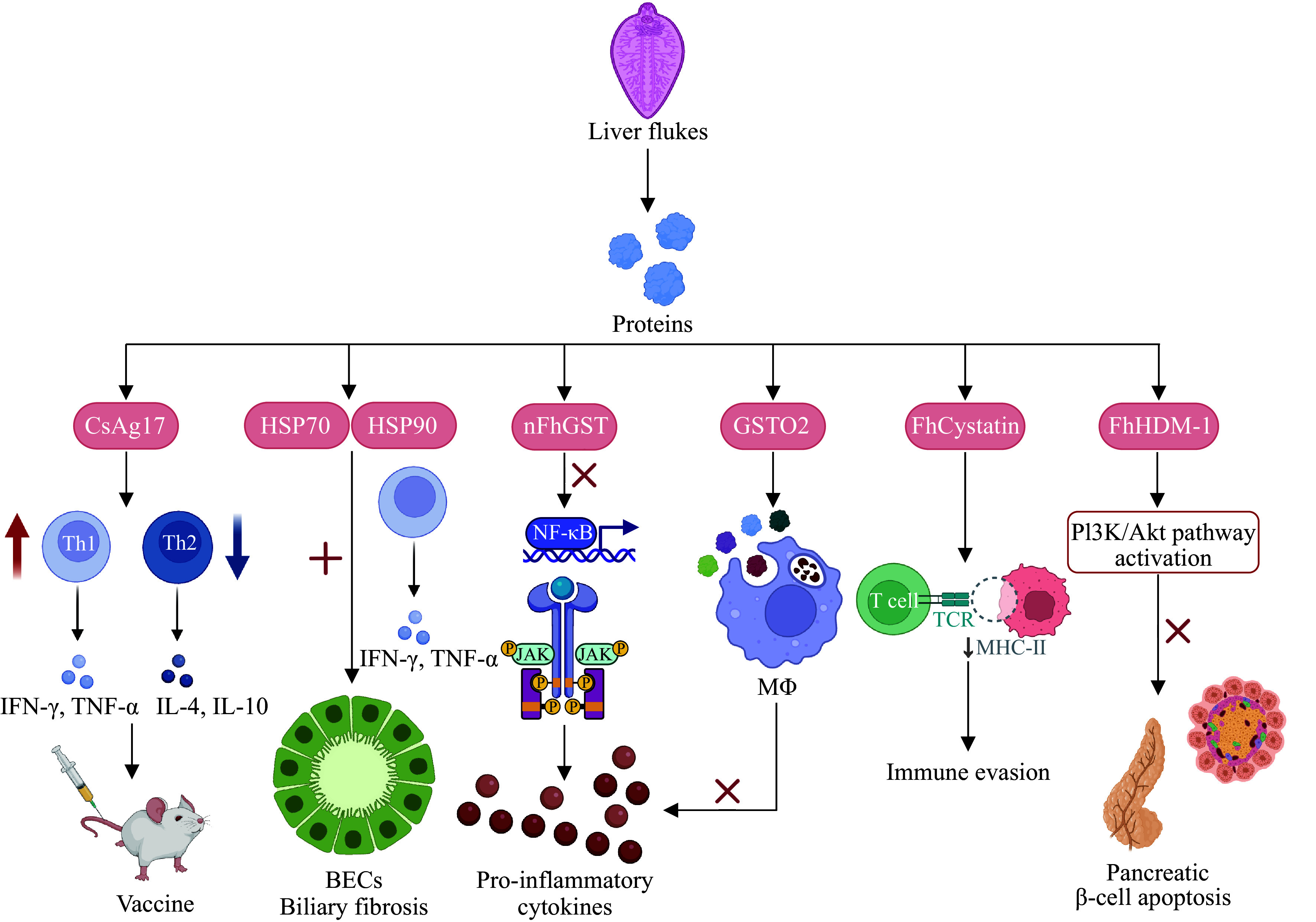
Immune and metabolic regulation by the liver fluke-derived molecules. Abbreviations: CsAg17, C. sinensis secretory protein CsAg17; nFhGST, native *F. hepatica* glutathione S-transferase; GSTO2, glutathione S-transferase Omega 2; BECs, biliary epithelial cells; FhHDM-1, *F. hepatica* helminth defense molecule 1; HSP70/90, *C. sinensis*-derived heat shock protein 70/90; NF-κB, nuclear factor kappa-B; IFN-γ, interferon-gamma; TNF-α, tumor necrosis factor-alpha; IL-4/10, interleukin-4/10; JAK, Janus kinase; TCR, T-cell receptor; MHC-Ⅱ, major histocompatibility complex class Ⅱ molecules; PI3K/Akt, phosphatidylinositol 3-kinase/protein kinase B.

The food-borne liver fluke *Opisthorchis felineus* [ *O. felineus* (Rivolta, 1884) Branchard, 1895] is an epidemiologically important parasite in humans, widespread in Eastern Europe, Russia, and Western Europe, which induces liver morbidities, such as periductal fibrosis, bile duct neoplasia, and chronic inflammation. One study discovered the capacity of *O. felineus* ESPs and EVs to stimulate the formation of pseudo-capillaries *in vitro*, which might be correlated with the liver fluke-induced pathogenesis and promote the discovery of new treatment targets to alleviate or prevent the most serious complications of opisthorchiasis ^[ 95] ^. Additionally, *O. felineus* ESPs and lysate might heal skin wounds by reducing inflammation, modulating vascular response, and stimulating collagen formation and extracellular matrix remodeling ^[ 96] ^. Finally, the regulation of host immunity and metabolism by various liver fluke-derived molecules is partially summarized in ***Fig. 2***.

## Nematode-derived molecules

### Proteases/protease inhibitors

*Trichinella spiralis* [ *T. spiralis* (Owen, 1835) Railliet, 1895], is the primary causative agent of trichinellosis, a serious zoonotic parasitosis caused by the intake of raw or undercooked meat containing larvae cysts. The *T. spiralis* derived-proteases play important roles in larval invasion and triggering local mucosal immune responses. For instance, a novel *T. spiralis* cathepsin L-like cysteine peptidase (TsCL) promoting the larval invasion of host intestinal epithelium cells induced pro-inflammatory responses, including macrophage M1 polarization *via* the NF-κB pathway ^[ 97] ^.

Meanwhile, protease inhibitors with strong immunomodulatory functions are promising in alleviating inflammatory diseases. *T. spiralis* serine protease inhibitors (TsSPIs) detected in the ESPs of muscle larvae and adult worms have been proven to have a similar function on the invasion of host intestinal epithelium cells and the vaccine potential as the above-mentioned protease ^[ 98] ^. TsSPIs also played therapeutic roles in inflammatory models such as inflammatory bowel disease and non-alcoholic fatty liver disease by activating M2 phenotypic macrophages and Tregs ^[ 99– 100] ^. *T. spiralis* cystatin (TsCys) derived from adult worms also alleviated inflammatory models like sepsis by activating M2 phenotypic macrophages mediated by inhibiting the TLR2/MYD88 signaling pathway ^[ 101] ^. Additionally, investigators identified a novel recombinant cystatin derived from muscle-stage *T. spiralis* (rTsCstN), and discovered that it elicited an anti-inflammatory activity by inhibiting pro-inflammatory cytokines and disrupting the antigen presentation pathway by reducing the expression level of major histocompatibility complex (MHC) class Ⅱ ^[ 102] ^.

*Nippostrongylus* *brasiliensis* ( *N. brasiliensis*, Travassos, 1914), a worldwide hookworm that naturally infects rats as well as mice, serves as a study model for hookworms to investigate the immune responses of the host. Cysteine proteases identified in the ESPs of *N. brasiliensis* preferentially evoked an IgE/IgG1 antibody response, promoting the design of modified allergen-vaccines from the perspective of cysteine proteases ^[ 103] ^. Moreover, nippocystatin, a cystatin derived from *N. brasiliensis*, played crucial roles in inhibiting antigen processing, modulating the antigen-specific immune response, and achieving immune evasion, which may be a potential vaccine candidate against *N. brasiliensis* infection ^[ 104] ^.

### Proteins/peptides

In addition to the oncogenic potential of *T. spiralis* in the formation of tumors, such as melanoma, oral squamous cell carcinoma, and other tumor types in animals and humans, its potential anticancer effects have also been thoroughly reviewed ^[ 105] ^. Similarly, the potential immunomodulatory properties of *T. spiralis* at the derived-molecule level have been reported. For instance, the recombinant *T. spiralis* derived-53-kDa protein (rTsP53) ameliorated the TNBS-induced colitis by promoting Th2 immune responses and M2 macrophage polarization ^[ 106] ^. Similar protection against inflammation was also shown by using the *T. spiralis-*derived paramyosin (TsPmy) in a DSS-induced colitis model, mediated by inducing thymic-derived Tregs and promoting the differentiation of effector Tregs with a higher suppressive function and stability in the inflamed colon ^[ 107] ^.

Smallwood *et al* ^[ 108]^ identified two peptides from the ESPs of two kinds of parasitic hookworms, *Ancylostoma caninum* ( *A. caninum*, Ercolani, 1859) 1 (Acan1) and *Necator americanus* ( *N. americanus*, Stile, 1902) 1 (Nak1), which significantly attenuated pathological damages, such as weight loss, colonic atrophy, edema, ulceration, and necrosis, in mice exposed to TNBS and showed better anti-colitis properties by inhibiting the proliferation of CD4 ^+^ T cells and the production of IL-2 and TNF. Two tissue inhibitors of metalloprotease (TIMP)-like proteins (Ac-TIMP-1 and Ac-TIMP-2), redesignated as anti-inflammatory protein-1 (AIP-1) and AIP-2, have been identified among *A. caninum* secreted proteins ^[ 109] ^, among which Ac-AIP-2 alleviated airway inflammation in a mouse model of asthma by inducing mesenteric regulatory DCs and Tregs ^[ 110] ^. Both Ac-AIP-1 and Ac-AIP-2 have been discovered to relieve the *Trypanosoma cruzi*-induced cardiac inflammation by suppressing cardiac cellular infiltration and reducing cardiac levels of interferon-gamma (IFN-γ), IL-6, and IL-2 ^[ 111] ^. Investigators also identified two proteins containing the netrin domain from the *N. americanus* secretome, one of which was named Na-AIP-1, and found that it inhibited the TNBS-induced colitis model in mice by reducing the secretion of cytokines related to Th1/Th17 immune responses and TNF in the intestine ^[ 112] ^. Thus, Na-AIP-1 is now a candidate for clinical drug development and as a potential novel therapeutic for immune-mediated diseases. In recent years, investigators have established Na-AIP-1 as a potential new molecule for the treatment of arthritis considering its therapeutic potential in collagen-induced arthritis ^[ 113] ^.

Investigators have also devoted themselves to mining some anti-inflammation and therapeutic molecules derived from *N. brasiliensis* by utilizing the multi-omics technology. Experiments both *in vivo* and *in vitro* showed that proteins derived from *N. brasiliensis* (mixed proteins) induced an M2-polarized phenotype and exhibited anti-inflammatory properties, which generated protective effects on the initial and progressive stages of atherosclerosis ^[ 114– 115] ^. Thuma *et al* ^[ 116]^ further identified an *N. brasiliensis* larval secreted-protein 1 (Nb-LSA1) from ESPs, and discovered that it elicited a strong IgG1 response and reduced larval migration to the lung in a basophil-dependent manner to protect against *N. brasiliensis* infection, which may be beneficial for the development of promising vaccination strategies.

### EVs/EV-loaded molecules

The EVs released from *T. spiralis* muscle larvae with immunomodulatory functions were found to prevent colitis, mediated by inhibiting the DSS-induced M1 macrophage polarization and the expression of pro-inflammatory cytokines ( *i.e.*, TNF-α, IFN-γ, IL-17A, and IL-1β), but promoting the infiltration of M2 macrophages and the expression of anti-inflammatory cytokines ( *i.e.*, IL-4, IL-10, TGF-β, and IL-13) into the colon ^[ 117] ^. *T. spiralis* muscle larvae EVs also ameliorated the colitis by decreasing the count of Th1/Th17 cells and increasing the count of Th2/Treg cells in the TNBS-induced colitis mice model ^[ 118] ^. Similarly, EVs derived from adult *N. brasiliensis* also suppressed pro-inflammatory cytokines ( *i.e.*, IL-6, IL-1β, IFN-γ, and IL-17a) but induced high levels of the anti-inflammatory cytokine IL-10 to protect against colitic inflammation in the gut of mice; while the whipworm *Trichuris muris* ( *T. muris*, Schrank, 1788) EVs did not have these anti-inflammatory properties, which was mainly correlated with the differences in their unique EVs-loaded miRNAs ^[ 119] ^. Additionally, compared with *N. brasiliensis* soluble ESP proteins, *N. brasiliensis*-EVs induced a unique cytokine profile (the higher levels of IL-10 secretion) in the colon, which also might be due to EVs-loaded miRNAs ^[ 119] ^. Remarkably, adult *N. brasiliensis*-EVs miRNAs might map to the interleukin network, especially IL-6 receptor and IL-6 signal transducers, IL-17 receptor genes, and IL-21, thereby achieving anti-inflammatory effects ^[ 119] ^.

### Antigens

Upon encountering pathogens, neutrophil extracellular traps, which capture pathogens and trigger phagocytosis and cytokine production, are one of the ways that polymorphonucleocytes release to eliminate pathogens. *T. spiralis* ESPs were found to inhibit phorbol 12-myristate 13-acetate-induced neutrophil extracellular trap generation by suppressing the production of reactive oxygen species and enhancing *Escherichia coli* engulfment by polymorphonucleocytes, which potentially impaired innate immune responses in the host and established chronic infections ^[ 120] ^. Moreover, the *T. spiralis*-derived mixed antigens (TsAg) were believed to be novel therapeutic agents for non-alcoholic fatty liver disease, which ameliorated hepatic inflammation by inhibiting pro-inflammatory TNF-α and IL-1β as well as promoting M2 macrophage polarization, and suppressed hepatic steatosis by improving insulin resistance as well as regulating the abnormal expression of hepatic lipid-related genes ^[ 121] ^. It was also found that TsAg attenuated colitis severity through M2 macrophage polarization mediated by promoting the expression of programmed death receptor 1 (PD-1) in the DSS-induced colitis model ^[ 122] ^. Additionally, TsAg conferred a therapeutic effect on sepsis-induced acute lung injury through activating Tregs and inhibiting pro-inflammatory cytokines *via* the high mobility group box 1/TLR2/MYD88 signaling pathway ^[ 123] ^. As a unique kind of inflammation modulator, the antitumor effect of TsAg is not negligible either, which may be mediated by directly inducing tumor cell apoptosis and indirectly inhibiting tumor cell growth through inducing Th1 immune responses in the early stage of *T. spiralis* infection ^[ 124] ^.

Among *N. brasiliensis* ESPs, some small molecules, including L-glutamine, glutamine (Gln), pyruvate, and alanine-Gln (Ala-Gln), identified through the metabolomics approach, may have anti-inflammatory properties, which need translational studies for evaluation and verification ^[ 125] ^. Khudhair *et al* ^[ 126]^ demonstrated that *N. brasiliensis* ESPs significantly reduced glucose tolerance and body weight in a mouse model of type 2 diabetes, likely mediated by the increase of eosinophilia and IL-5 in peripheral tissues, showing its therapeutic potential for treating some metabolic disorders.

## Cestode-derived molecules

### Proteases/protease inhibitors/redox enzymes

Cystic echinococcosis caused by *Echinococcus granulosus* ( *E. granulosus*, Batsch, 1786) is a common zoonotic parasitic disease with a global distribution, posing a serious threat to human health and a significant financial burden on the society. Neutrophil elastase released by the activated neutrophils also plays significant roles in the process of inflammation and cancer metastasis ^[ 29, 127] ^. The Kunitz-type serine protease inhibitor family identified in *E. granulosus* oncospheres (EgKI-1 and EgKI-4) has shown potential anti-cancer properties ^[ 128] ^. By inhibiting neutrophil elastase, EgKI-1 significantly reduced neutrophil inflammatory infiltration in a carrageenan-induced mouse air pouch model of local inflammation. This suggests EgKI-1 may have the potential to act as a novel anti-inflammatory therapeutic ^[ 128] ^. Sagasti *et al* ^[ 129]^ reported that both EgKU-1 and EgKU-4, Kunitz-type inhibitors of the voltage-activated cation channels (K _v_), inhibited the production of Th1/Th17 cytokine subunit IL-12/23p40 by macrophages stimulated with the TLR4 agonist LPS. Additionally, EgKU-4, but not EgKU-1, inhibited the production of inflammatory cytokine IL-6 ^[ 129] ^. Therefore, further research is necessary for a more thorough understanding of the activities of different Kunitz-type inhibitor families against inflammatory cytokines and the underlying mechanisms.

In addition, TPx secreted by *E. granulosus* protoscoleces (EgTPx) was crucial for the survival of protoscoleces and involved in the antioxidant defense against the host throughout development, revealing a promising strategy for creating novel anti-echinococcosis medications ^[ 130] ^. Consistent with the immunoregulatory roles of rSjTPx, EgTPx also induced macrophage recruitment and M2 polarization *via* the PI3K/AKT/mammalian target of rapamycin pathway, which might play an essential role in the regression of inflammation ^[ 131] ^.

### Proteins/peptides

A zoonotic parasitic disease known as alveolar echinococcosis is caused by the infection with metacestode larvae of the tapeworm *Echinococcus multilocularis* ( *E. multilocularis*, Leuckart, 1863). Considering the limited effectiveness of traditional chemotherapy and the urgent need for novel prophylactic and therapeutic approaches in the treatment of alveolar echinococcosis, helminth therapy has attracted the attention of investigators ^[ 132] ^. With the growing application of bioengineered nano cellular membrane vesicles to display the native conformational epitope peptides, the dominant epitope peptides of four T-cells and four B-cells derived from *E. multilocularis* with high immunogenicity were engineered into the Vero cell surface, which activated DCs, induced specific T/B cells to form a mutually activated circuit, and effectively inhibited *E. multilocularis* infection ^[ 132] ^. A factor with structural and functional homologies to mammalian activin A has been identified from *E. multilocularis* ESPs, named *E. multilocularis* activin A homolog (EmACT). This factor was found to induce the secretion of IL-10 by T-cells and contributed to the expansion of TGF-β-driven Foxp3 ^+^ Treg ^[ 133] ^.

It has also been demonstrated that molecules derived from *Taenia crassiceps* ( *T. crassiceps*, Zeder, 1800) have antitumor-immune effects on the host. CD8 ^+^ T cells were found to kill malignant cells in an antigen-specific manner. The *T. crassiceps*-derived peptide glycerol kinase-1 was found to treat cancer by promoting CD8 ^+^ T cell responses in a murine model of melanoma, mediated by suppressing the expression of PD-1 on tumor-infiltrating CD8 ^+^ T cells and by reducing the high expression levels of tumor-induced PD-1 ligand 1 on the surface of DCs ^[ 134] ^.

### EVs/EV-loaded molecules

EVs secreted from *E. granulosus* protoscolex culture supernatant (PCS) and hydatic cyst fluid (HCF) are internalized by CD4 ^+^ T cells, CD8 ^+^ T cells, B cells, DCs, and other immune cells. However, the capacity of the same type of immune cells to uptake PCS-EVs and HCF-EVs varies. Jeong *et al* ^[ 135]^ used EVs extracted from *E. granulosus* HCF to act on mouse lung epithelial cells, finding that these EVs internalized by mouse lung epithelial cells alleviated Th2 allergic airway inflammation by inducing Tregs. According to different centrifugal forces (2000 *g*,10000 *g*, and 110000 *g*), both the *E. granulosus* HCF- and PCS-EVs were divided into 2 K, 10 K, and 110 K EVs, respectively. These categories showed biological differences in the regulation of host immunity and metabolism. Among them, HCF 110 K EVs might be internalized by sheep peripheral blood mononuclear cells in a time-dependent manner, thereby increasing the expression levels of IL-10, TNF-α, and IRF5, but significantly decreasing the expression levels of IL-1β, IL-17, and CD14; however, PCS 110 K EVs were more easily internalized by hepatocytes than both 10 K and 2 K EVs ^[ 136– 137] ^. Interestingly, the same helminth EVs have different protein and miRNA compositions at different infection stages, whose regulatory effects on host immunity and metabolism may also be different. Some investigators analyzed the immunoregulatory functions of plasma-derived EVs from mice with *E. granulosus* infection at different infection stages. The co-culture experiments of EVs and mononuclear lymphocytes showed that compared with the 0W-EVs (collected 0 weeks post-infection), the 7W-EVs induced the relative abundance of Tregs, increased the expression level of IL-10, but decreased the related cytokines secreted by Th cells; compared with the 0W-EVs, the 20W-EVs significantly decreased the levels of IFN-γ and IL-6; compared with the 7W-EVs, the 20W-EVs decreased the levels of IL-6 and IL-10, but significantly increased the levels of IL-17A and IL-2; these results indicated the different degrees of internalization of the plasma derived-EVs from mice with *E. granulosus* infection by immune cells at different infection stages ^[ 138] ^.

### Antigens

Soluble antigens secreted by *E. granulosus* were found to promote M2 polarization *via* the ras homolog family member A-MAPK pathway, and then stimulated the proliferation and transformation of HSCs, establishing a long-term infection and eventually leading to liver fibrosis ^[ 139] ^. Antigen B secreted by the larva *E. granulosus* acted as a potential receptor to bind to tissue-resident macrophages, recruiting inflammatory monocytes during infection, which induced an anti-inflammatory phenotype in macrophages ^[ 140] ^. The *E. granulosus*-secreted HCF, germinal layer antigen, PCS, and other products have enormous significance and development prospects in the regulation of host immunity and metabolism. For instance, *E. granulosus* ESPs alleviated dysbiosis of the gut microbiota and was used to treat cognitive impairment induced by a high-fat diet by suppressing colon inflammation, neuroinflammation, and activation of the microglia and astrocytes, which may be a promising drug candidate against obesity-related neurodegenerative diseases ^[ 141] ^. Vaccination with *E. granulosus* antigens extracted from the HCF of *E. granulosus*-infected patients might mediate anti-tumor immunity against colon cancer and non-small cell lung cancer in mice by promoting the activation of host NK cells ^[ 142– 143] ^. Glycomolecules in *E. granulosus* HCF even interfered with the TLR4-mediated DC activation by regulating the C-Raf protooncogene, serine/threonine kinase (cRAF) phosphorylation pathway in the bone marrow-derived DCs, leading to the downregulation of signal transduction of CD80, CD86, NF-κB, and p38MAPK as well as downstream inflammatory factors TNF-α and IL-12 ^[ 144] ^. To better investigate the specific molecules in HCF that play important roles in the regulation of host immunity and metabolism, the separation and identification of HCF molecules will also be a research direction in the future.

In line with the glycomolecules in *E. granulosus* HCF, *T. crassiceps* ESPs, through their glycomolecules, also inhibited TLR-mediated DC maturation and secretion of IL-12 and TNF-α in a cRAF-dependent pathway, thereby eliciting a Th2 immune response, which demonstrated a new intracellular mechanism of the immunomodulatory potential ^[ 145] ^. Glycosylated molecules derived from helminths have been suspected to play an important role in triggering Th2 responses, whereas the correlation of cRAF phosphorylation pathway with anti-inflammatory or inflammatory DC responses remains unknown and needs future studies ^[ 146] ^. Additionally, Callejas *et al* ^[ 147]^ reported that the *T. crassiceps* ESPs reduced the production of inflammatory cytokines, such as IL-1β, TNF-α, IL-33, and IL-17, and significantly suppressed colon carcinogenesis in the colitis-associated colon cancer mice model, which might be related to the inhibition of the STAT3/NF-κB signaling pathway.

## Conclusions

**Figure 3:**
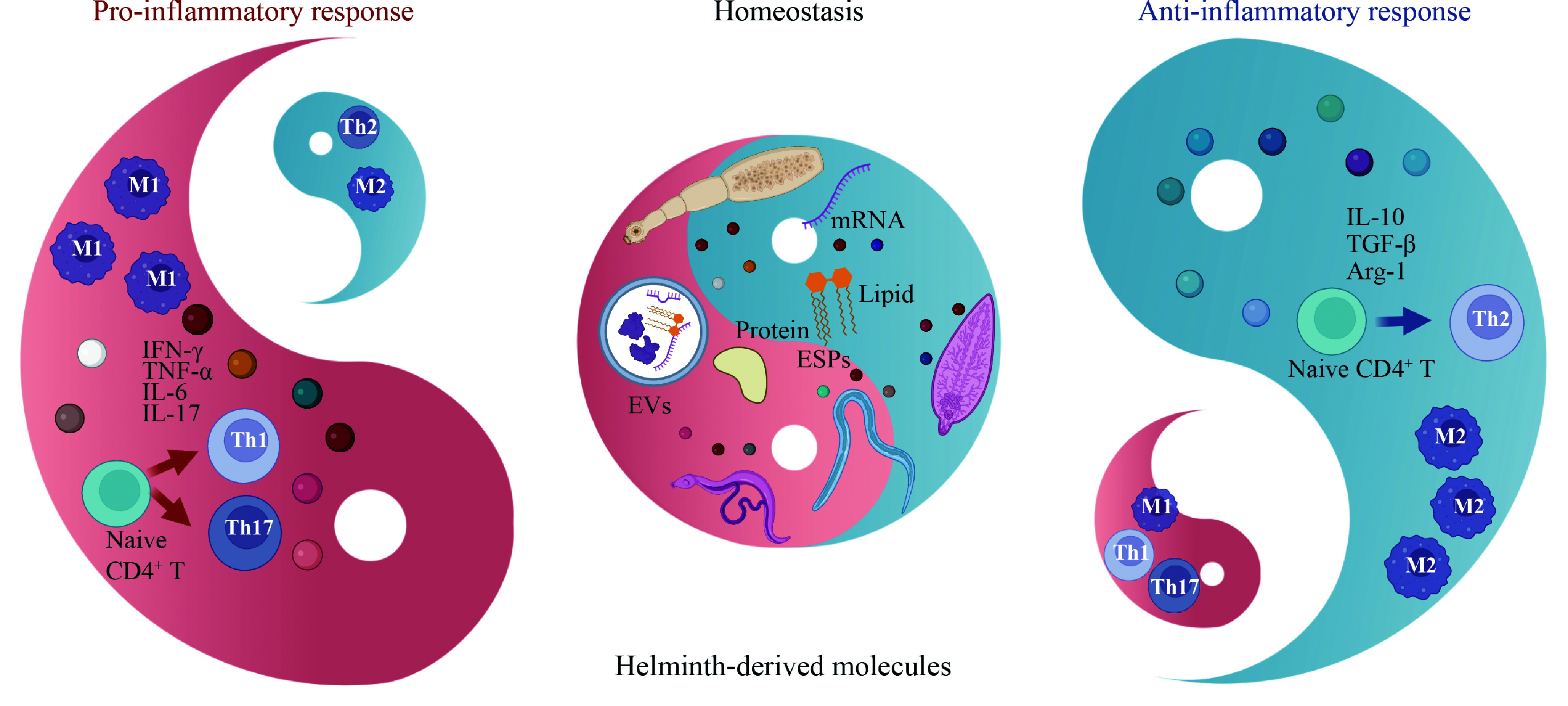
"Double-edged sword" roles of helminth-derived molecules in host immune homeostasis. Abbreviations: ESPs, excretory/secretory products; EVs, extracellular vesicles; M1, classical activated macrophages; M2, alternatively activated macrophages; Th1, T helper 1 cells; Th2, T helper 2 cells; Th17, T helper 17 cells; IFN-γ, interferon-gamma; TNF-α, tumor necrosis factor-alpha; IL-6/7/10, interleukin-6/7/10; TGF-β, transforming growth factor-beta; Arg-1, arginase-1; CD4, cluster of differentiation 4.

Considering the acceleration of globalization and increasingly frequent international exchanges, such as large-scale Chinese governmental programs "Aid to Africa" and "Belt and Road Initiative", the imported parasitic diseases occur from time to time and are increasing day by day. Thus, the prevention and treatment of parasitic diseases in our nation are facing both significant opportunities and challenges. Through in-depth investigation of the pathogenic and pharmacopeial roles (pathogenic mechanisms as well as immunity and metabolism regulation) of essential helminth-derived molecules, investigators are committed to exploring new biomarkers and targets for the prevention and control of parasitic diseases. This current review on the special composition and functional properties of helminth-derived molecules may prove crucial to the understanding of host-parasite coevolution and host immune homeostasis regulation ( ***Fig. 3***). Helminth-derived molecules are promising for discovering more translational applications and pharmacological values on host immunity and metabolism in the future. Similarly, it is conceivable to develop a new approach for identifying and treating a range of immune diseases by exploring the potential immunopharmacological mechanisms of helminth-derived molecules. Most of the above-mentioned "double-edged sword" roles of helminth-derived molecules on the host have not yet been clarified completely. Therefore, we have reviewed recent progress on this topic and summarized key findings in ***Table 1*** to uncover and clarify the profound effect of helminth molecules on the development of parasitology, the diagnosis and treatment of parasitic diseases, and even many other diseases.

**Table 1:** Immune and metabolic regulation by the helminth-derived molecules

Species	Characteristic	Molecules	Regulation on the host
*Schistosoma japonicum*	Proteases	*S. japonicum* cathepsin B2 (SjCB2)	Facilitating cercariae invasion through the skin in schistosome infection ^[ 23] ^.
	Protease inhibitors	*S. japonicum* cysteine protease inhibitor (Sj-Cys)	Treating sepsis through alternatively activated macrophages (M2) ^[ 24] ^. Mitigating sepsis-induced cardiomyopathy through inhibiting the lipopolysaccharides (LPS)-myeloid differentiation primary response 88 (MYD88) inflammatory signaling pathway ^[ 25] ^. Presenting a protective effect against acute kidney injury induced by acute liver failure through inhibiting pyroptosis and nuclear factor kappa-B (NF-κB) signaling pathway ^[ 26] ^. Attenuating atherosclerotic renal damage through promoting regulatory T (Treg) and M2 macrophage polarization, mediated by inhibiting the TLR2/MYD88 signaling pathway ^[ 27] ^. Conferring a protective role in the mouse model of bone metabolic disorders by inhibiting the NF-κB signaling pathway, which is crucial for the early stages of osteoclastogenesis ^[ 28] ^.
	Redox enzymes	Recombinant *S. japonicum* thioredoxin peroxidases (rTPxs)	Exerting anti-inflammatory effects by triggering the production of M2 macrophages in response to LPS stimulation ^[ 33– 34] ^.
	Proteins	Heat shock protein 40 (Sjp40)	Stimulating the production of macrophages (MΦ), dendritic cells (DCs), and eosinophilic granulocytes (ECs) in non-parenchymal mouse liver cells, and promoting T helper 1 (Th1), Th2, and Th17 immune responses *in vitro* ^[ 35] ^. Activating the AMP-activated protein kinase (AMPK) pathway and subsequently attenuating lipogenesis ^[ 36] ^. Inhibiting the activation of hepatic stellate cells and expression of collagen 1α1 (COL1α1) in the activated HSCs to prevent and alleviate hepatic fibrosis ^[ 44– 45] ^.
	Proteins	Sjp90α	Inducing DCs in non-parenchymal mouse liver cells and Th17 immune response *in vitro* ^[ 35] ^.
	Proteins	Sjp60	Inducing Tregs and reducing liver immunopathology in mice with *S. japonicum* infection ^[ 37] ^.
	Proteins	Sjp60-derived peptide SJMHE1	Decreasing Th1/Th17 cells and increasing Th2/Treg cells to exert a protective effect on autoimmune diseases ^[ 38- 40] ^.
	Proteins	Recombinant *S. japonicum* excretory/secretory protein Sj16	Inhibiting dextran sodium sulfate-induced colitis in mice model ^[ 41] ^.

**Table 1:** Immune and metabolic regulation by the helminth-derived molecules (Continued)

Species	Characteristic	Molecules	Regulation on the host
	miRNAs	miR-2162-3p	Promoting the activation of HSCs and hepatic fibrosis in the host ^[ 48] ^.
	miRNAs	Sja-miR-1	Upregulating the expression levels of collagens and α-smooth muscle actin (α-SMA) as well as promoting schistosomiasis hepatic fibrosis ^[ 50] ^.
	miRNAs	Sja-miR-7-5p	Conferring an antitumor activity on hepatocellular carcinoma during schistosome infection by inhibiting the expression of S-phase kinase-associated protein 2 ^[ 51] ^.
	EVs	miR-125b and bantam miRNA	Regulating the respective targets (including prostate-specific antigen 1, family with sequence similarity 212 member B, and CXADR-like membrane protein) to promote the proliferation of macrophage and production of TNF-α ^[ 52] ^.
	EVs	miRNA-33	Upregulating the expression levels of α-SMA and COL1α1 *via* the transforming growth factor-β (TGF-β)/small mother against decapentaplegic family member 3 (SMAD3) signaling pathway in liver tissues and leading to hepatic fibrosis ^[ 53] ^.
	EVs	Sja-miR-71a	Suppressing the pathological progression of liver fibrosis by inhibiting TGF-β1/SMAD and interleukin-13 (IL-13)/signal transducer and activator of transcription 6 (STAT6) pathways *via* directly targeting semaphorin 4D ^[ 54] ^.
	EVs	Sja-miR-3096	Mediating antitumor activity in liver cancer through targeting phosphoinositide 3-kinase class II alpha ^[ 55] ^.
	Mixed antigens	Soluble egg antigen (SEA)	Promoting the production of M2 and the formation of hepatic immunopathology ^[ 62] ^. Suppressing rejection and prolonging skin graft survival by inhibiting Th1 immune responses and inducing Th2/Treg immune responses in skin transplantation ^[ 62] ^. Protecting against Type 2 Diabetes (T2D) by inhibiting Th1/Th17 immune responses and enhancing Th2/Treg immune responses ^[ 62] ^. Inducing the restoration of Treg/Th17 balance and inhibiting the glycolysis pathway and lipogenesis, which may play a protective role against trinitrobenzene sulfonic acid (TNBS)-induced colitis ^[ 62] ^. Alleviating ovalbumin-induced allergic airway inflammation ^[ 62] ^.
*Schistosoma mansoni*	Protease inhibitors	*S. mansoni* Kunitz-type serine protease inhibitor (SmKI-1)	Conferring an anti-inflammatory effect in acetaminophen-mediated liver damage, gout arthritis, and carrageenan-induced pleurisy model through inhibiting the activity of neutrophil elastase and neutrophil inflammatory infiltration ^[ 30] ^. Maintaining a Th1/Th2-type balanced protective response and producing a solid IgG response against *S. mansoni* infection ^[ 31] ^.
	Peptides	Two synthetic peptides of Sm16 (KS-84 and KS-66)	KS-84 might inhibit the activation of HSCs by reducing TGF-β1 signaling, while KS-66 might enhance the activation of HSCs ^[ 46] ^.
	EVs	miR-10	Downregulating NF-κB activity through targeting mitogen-activated protein kinase 7 (MAPK7) in Th cells ^[ 56] ^.
	EVs	Surface glycans	Enhancing the expression of IL-12 and IL-10 in DCs ^[ 58] ^. Participating in immune evasion by being coated with sialoglycans *in vivo* or being encapsulated by exogenous host sialylated molecules ^[ 60] ^.
	Mixed antigens	SEA	Improving wound healing in a variety of wound models by inducing Th2/Tregs and decreasing the expression of IL-17A in gamma delta (γδ) T cells ^[ 60] ^. Preventing T2D in NOD mice by enhancing Th2/Treg immune responses ^[ 60] ^.
*Clonorchis sinensis*	Proteases	Cysteine proteases	Facilitating Th2/Treg immune responses ^[ 69] ^.
	Proteins	HSP70/90	Inducing strong Th1 immune responses and promoting the secretion of pro-inflammatory cytokines in the mouse biliary epithelial cells and the progression of clonorchiasis ^[ 79– 80] ^.
	Proteins	CsAg17 protein	Alleviating the helminth burden and have the potential to serve as a good vaccine candidate against *C. sinensis* infections ^[ 81] ^.
	EVs	miRNA Csi-let-7a-5p	Promoting the M1 (classically activated macrophages) polarization and contributing to the biliary injuries through targeting the suppressor of cytokine signaling 1- and C-type lectin domain family 7 member A-mediated NF-κB signaling pathway ^[ 84] ^.

**Table 1:** Immune and metabolic regulation by the helminth-derived molecules (Continued)

Species	Characteristic	Molecules	Regulation on the host
	EVs	Csi-miR-96-5p	Inhibiting ferroptosis by regulating the phosphatase and tensin homolog/solute carrier family 7 member 11/glutathione peroxidase 4 axis, thereby promoting cholangiocarcinoma (CCA) proliferation and migration ^[ 86] ^.
	Mixed antigens		Promoting the activation of HSCs and pathogenesis of hepatic fibrosis *via* TLR4 and TGF-β/SMADs signaling pathway ^[ 90] ^. Promoting the production of IL-6 in mouse biliary epithelial cells through TLR2-mediated protein kinase B (AKT) and p38 pathways, resulting in the aggravation of hepatic fibrosis in mice ^[ 91] ^. Promoting the progression of CCA through the interaction between normal cholangiocytes and CCA cells, mediated by extracellular signal-regulated kinase 1/2/NF-κB/matrix metalloproteinase-9 and integrin β4-focal adhesion kinase/steroid receptor coactivator ^[ 93– 94] ^.
*Fasciola hepatica*	Proteases	Cathepsin L-like cysteine peptidases	Inducing the alternative activation of murine DCs NLR family pyrin domain containing 3 inflammasome and the production of pro-inflammatory cytokines (IL-1β and IL-18) ^[ 70] ^.
	Protease inhibitors	Kunitz-type serine protease inhibitor (FhKTM)	Promoting Th2 immune responses and inhibiting Th1/Th17-associated inflammatory responses ^[ 72] ^.
	Protease inhibitors	Cystatins (FhCystatin)	Regulating the activity of macrophages, participating in immune evasion, and preventing excessive damage to both parasite and host tissues ^[ 73] ^.
	Redox enzymes	Native glutathione S-transferase (nFhGST)	Conferring anti-inflammatory effects on the host through targeting multiple effectors of NF-κB, JAK/STAT signaling, and other pathways and inhibiting the production of pro-inflammatory cytokines/chemokines ^[ 76– 77] ^.
	Redox enzymes	A novel glutathione S-transferase Omega 2 (GSTO2)	Promoting apoptosis and inhibiting the production of pro-inflammatory cytokines ^[ 78] ^.
	Peptides	Helminth defence molecule 1 (FhHDM-1)	Activating phosphatidylinositol-3-kinase (PI3K)/AKT pathway to prevent apoptosis of pancreatic β-cells and then treat T1D in mice ^[ 83] ^.
*Opisthorchis viverrini*	EVs		Promoting the proliferation of bile duct cells and the secretion of IL-6, which exerts a tumorigenic phenotype ^[ 87] ^.
	EVs	Recombinant tetraspanin 2 (recombinant- *O. viverrini*-tetraspanin-2, rOv-TSP-2) and rOv-TSP-3	Inducing antibody responses that block the EVs from being phagocytized by bile duct cells and reducing the parasite burden ^[ 88] ^.
*Opisthorchis felineus*	Mixed antigens		Stimulating the formation of pseudo-capillaries *in vitro* ^[ 95] ^. Heal skin wounds by reducing inflammation, modulating vascular response, and stimulating collagen formation and extracellular matrix (ECM) remodeling ^[ 96] ^.
*Trichinella spiralis*	Proteases	Cathepsin L-like cysteine peptidase (TsCL)	Promoting the process of *T. spiralis* larval invasion of intestinal epithelium cells (IECs) and inducing pro-inflammatory responses including macrophage M1 polarization *via* the NF-κB pathway ^[ 97] ^.
	Protease inhibitors	Serine protease inhibitor (TsSPI)	Promoting the process of *T. spiralis* larval invasion of host IECs and having vaccine potential as the above protease ^[ 98] ^. Playing therapeutic roles in inflammatory models like inflammatory bowel disease and non-alcoholic fatty liver disease through activating M2 phenotypic macrophages and Tregs ^[ 99– 100] ^.
	Protease inhibitors	Cystatin (TsCys)	Alleviating sepsis through activating M2 phenotypic macrophages mediated by inhibiting the TLR2/MYD88 signaling pathway ^[ 101] ^.
	Protease inhibitors	A novel recombinant cystatin derived from muscle-stage (rTsCstN)	Eliciting an anti-inflammatory activity by inhibiting pro-inflammatory cytokines and disrupting the antigen presentation pathway by reducing the expression of major histocompatibility complex (MHC) class Ⅱ ^[ 102] ^.
	Proteins	53-kDa protein (TsP53)	Ameliorating TNBS-induced colitis through promote Th2 immune responses and M2 macrophage polarization ^[ 106] ^.

**Table 1:** Immune and metabolic regulation by the helminth-derived molecules (Continued)

Species	Characteristic	Molecules	Regulation on the host
	Proteins	Paramyosin (TsPmy)	Eliciting an anti-inflammatory activity in dextran sodium sulfate-induced colitis model, mediated by inducing thymic-derived Tregs and promoting the differentiation of effector Tregs with higher suppressive function and stability in the inflamed colon ^[ 107] ^.
	EVs		Preventing colitis by inducing M2, Th2 and Treg cells-mediated immune responses and inhibiting M1, and Th1/17 cells-mediated immune responses ^[ 117– 118] ^.
	Mixed antigens		Inhibiting phorbol 12-myristate 13-acetate-induced neutrophil extracellular traps generation through suppressing the production of reactive oxygen species and enhancing *Escherichia coli* engulfment by polymorphonucleocytes ^[ 120] ^. Ameliorating non-alcoholic fatty liver disease by suppressing hepatic inflammation and steatosis ^[ 121] ^. Attenuating colitis severity through enhancing M2 macrophage polarization, mediated by promoting the expression of programmed death receptor 1 ^[ 122] ^. Conferring a therapeutic effect on sepsis-induced acute lung injury through activating Tregs and inhibiting pro-inflammatory cytokines through high mobility group box 1/TLR2/MYD88 signaling pathway ^[ 123] ^. Promoting tumor cell apoptosis and inhibiting tumor cell growth through inducing Th1 immune responses in the early stage of *T. spiralis* infection ^[ 124] ^.
*Nippostrongylus brasiliensis*	Proteases	Cysteine proteases	Evoking an IgE/IgG1 antibody response ^[ 103] ^.
	Protease inhibitors	Nippocystatin	Inhibiting antigen processing, modulating antigen-specific immune response, and achieving immune evasion ^[ 104] ^.
	Mixed proteins		Inducing an M2-polarized phenotype and exhibiting anti-inflammatory properties in the initial and progressive stages of atherosclerosis (AS) ^[ 114– 115] ^.
	Proteins	Larval secreted-protein 1 (Nb-LSA1)	Eliciting a strong IgG1 response and reducing larval migration to the lung in a basophil-dependent manner to protect against *N. brasiliensis* infection ^[ 116] ^.
	EVs		Suppressing pro-inflammatory cytokines (IL-6, IL-1β, IFN-γ, and IL-17a) and inducing high levels of the anti-inflammatory cytokine IL-10 to protect against colitic inflammation in the mice ^[ 119] ^. Mapping to the interleukin network to modulate the anti-inflammatory response ^[ 119] ^.
	Small molecules: L-glutamine, glutamine (Gln), pyruvate, and alanine-Gln (Ala-Gln)		Having anti-inflammatory properties ^[ 125] ^.
	Mixed antigens		Reducing glucose tolerance and body weight in a mouse model of T2D, likely mediated by the increase of eosinophilia and IL-5 in peripheral tissues ^[ 126] ^.
*Ancylostoma caninum* and *Necator americanus*	Peptides	*A. caninum* 1 (Acan1) and *N. americanus* 1 (Nak1)	Attenuating pathological damage in mice exposed toTNBS ^[ 108] ^.
	Proteins	*A. caninum* anti-inflammatory protein-2 (Ac-AIP-2)	Alleviating airway inflammation in a mouse model of asthma by inducing mesenteric regulatory DC and Tregs ^[ 110] ^.
	Proteins	Ac-AIP-1 and Ac-AIP-2	Relieving *Trypanosoma cruzi*-induced-cardiac inflammation through suppressing cardiac cellular infiltration and reducing cardiac levels of IFN-γ, IL-6, and IL-2 ^[ 111] ^.
	Proteins	*N. americanus* anti-inflammatory protein-1 (Na-AIP-1)	Inhibiting TNBS-induced colitis model in mice by reducing the secretion of cytokines related to Th1/Th17 immune responses and TNF in the intestine ^[ 112] ^. Acting as a potential new molecule for the treatment of arthritis considering its therapeutic potential in collagen-induced arthritis ^[ 113] ^.

**Table 1:** Immune and metabolic regulation by the helminth-derived molecules (Continued)

Species	Characteristic	Molecules	Regulation on the host
*Echinococcus granulosus*	Protease inhibitors	Kunitz-type serine protease inhibitor (EgKI-1 and EgKI-4)	Having potential anti-cancer properties ^[ 128] ^.
	Proteins	EgKI-1	Reducing neutrophil inflammatory infiltration in carrageenan induced-mouse air pouch model of local inflammation ^[ 128] ^.
	Proteins	EgKU-1 and EgKU-4	Inhibiting the production of Th1/Th17 cytokine subunit IL-12/23p40 by macrophages stimulated with the TLR4 agonist LPS, and EgKU-4, but not EgKU-1 inhibiting the production of inflammatory cytokine IL-6 ^[ 129] ^.
	Redox enzymes	EgTPx	Promoting the survival of protoscoleces and the antioxidant defense against the host throughout development ^[ 130] ^. Inducing macrophage recruitment and M2 polarization *via* the PI3K/AKT/mammalian target of rapamycin pathway ^[ 131] ^.
	EVs	EVs extracted from *E. granulosus* HCF	Alleviating Th2 allergic airway inflammation through inducing Treg cells ^[ 135] ^.
	Mixed antigens		Promoting M2 polarization and the occurrence of liver fibrosis ^[ 139] ^. Acting as a potential receptor to bind to tissue-resident macrophages and recruiting inflammatory monocytes during infection, then inducing an anti-inflammatory phenotype in macrophages ^[ 140] ^. Alleviating dysbiosis of the gut microbiota and treating cognitive impairment induced by a high-fat diet ^[ 141] ^. Inducing antitumor immune response ^[ 142– 143] ^. Inhibiting TLR4-mediated inflammatory signaling pathway through regulating the C-Raf protooncogene, serine/threonine kinase (cRAF) phosphorylation pathway ^[ 144] ^.
*Echinococcus multilocularis*	Peptides	T-cells and B-cells dominant epitope peptides	Activating DCs, inducing specific T/B cells to form a mutually activated circuit, and then inhibiting *E. multilocularis* infection effectively ^[ 132] ^.
	Peptides	Activin A homolog (EmACT)	Inducing the secretion of IL-10 by T-cells and contributing to the expansion of TGF-β-driven Foxp3 ^+^ Treg ^[ 133] ^.
*Taenia crassiceps*	Peptides	Glycerol kinase-1	Promoting CD8 ^+^ T cell response in a murine model of melanoma to treat cancer ^[ 134] ^.
	Mixed antigens		Inhibiting TLR-mediated DC maturation and secretion of IL-12 and TNF-α in a cRAF-dependent pathway, thereby resulting in the Th2 immune response ^[ 145] ^. Reducing the production of inflammatory cytokines such as IL-1β, TNF-α, IL-33, and IL-17 and significantly suppressing colon carcinogenesis in the colitis-associated colon cancer (CAC) mice model, which may be related to the inhibition of the STAT3/NF-κB signaling pathway ^[ 147] ^.

Remarkably, it is not unusual to find molecules with commonalities in the regulation of the immunity and metabolism among helminths of different species, genera, or phyla, which may be attributed to the same components (like both *S. japonicum*-derived thioredoxin peroxidases SjTPx and *E. granulosus*-derived thioredoxin peroxidases EgTPx inducing M2 polarization), similar properties of different components (like both *T. crassiceps*-derived peptide glycerol kinase-1 and *E. granulosus*-derived Kunitz-type serine protease inhibitor family EgKI have potential anti-cancer properties), and the activation of the same signaling pathway (like both *S. japonicum*-derived cystatin Sj-Cys and the heat shock protein Sjp60 activating the TLRs/MYD88 signaling pathway). This is also a fascinating topic that will probably be worth reviewing further in the future.
